# Recording Strategies for High Channel Count, Densely Spaced Microelectrode Arrays

**DOI:** 10.3389/fnins.2021.681085

**Published:** 2021-07-13

**Authors:** Norberto Pérez-Prieto, Manuel Delgado-Restituto

**Affiliations:** Institute of Microelectronics of Seville (IMSE-Centro Nacional de Microelectrónica), Spanish National Research Council, Seville, Spain

**Keywords:** neuroscience, neural recording, time multiplexing, crosstalk, CMOS technology, prosthetics

## Abstract

Neuroscience research into how complex brain functions are implemented at an extra-cellular level requires *in vivo* neural recording interfaces, including microelectrodes and read-out circuitry, with increased observability and spatial resolution. The trend in neural recording interfaces toward employing high-channel-count probes or 2D microelectrodes arrays with densely spaced recording sites for recording large neuronal populations makes it harder to save on resources. The low-noise, low-power requirement specifications of the analog front-end usually requires large silicon occupation, making the problem even more challenging. One common approach to alleviating this consumption area burden relies on time-division multiplexing techniques in which read-out electronics are shared, either partially or totally, between channels while preserving the spatial and temporal resolution of the recordings. In this approach, shared elements have to operate over a shorter time slot per channel and active area is thus traded off against larger operating frequencies and signal bandwidths. As a result, power consumption is only mildly affected, although other performance metrics such as in-band noise or crosstalk may be degraded, particularly if the whole read-out circuit is multiplexed at the analog front-end input. In this article, we review the different implementation alternatives reported for time-division multiplexing neural recording systems, analyze their advantages and drawbacks, and suggest strategies for improving performance.

## 1. Introduction

**Figure 1:**
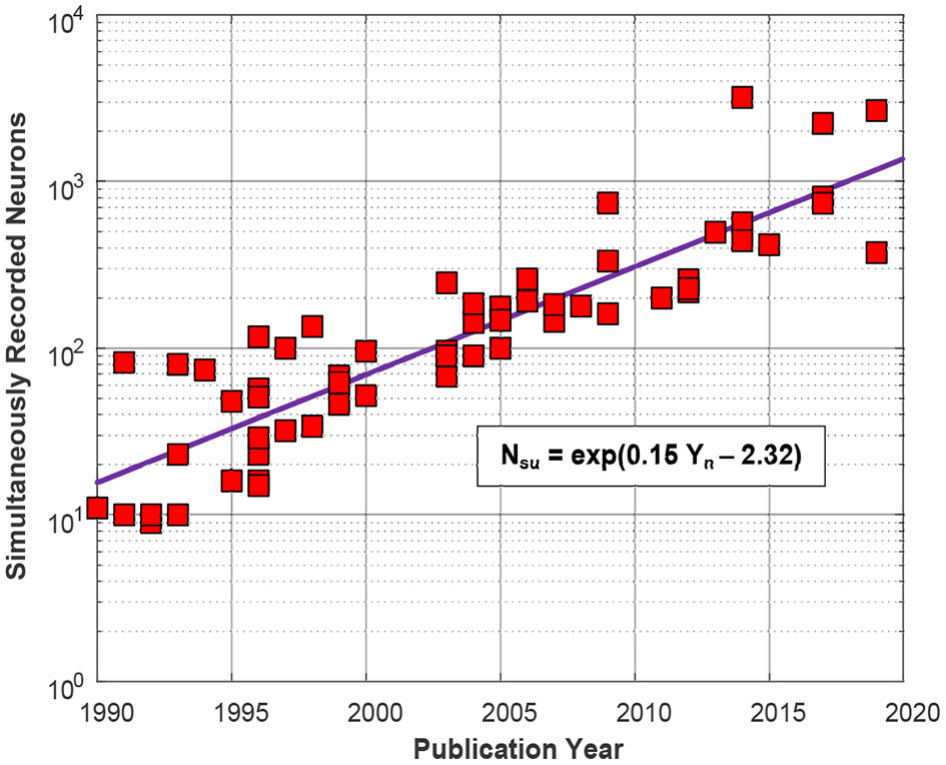
Number of simultaneously recorded neurons, *N*_*su*_, over the last three decades from a selection of *N* = 70 published works. Variable *Y*_*n*_ is the ordinal number of the years. The coefficient of determination of the fitting model is *R*^2^ = 84%.

One of the major challenges in neurophysiology is to identify the effective connectivity within the brain and reveal the subjacent drive-response map of the neural system (Friston, 2011; Sakkalis, 2011). This could help to understand the functional mechanisms underlying many neurological disorders which currently do not have effective treatments (Swann et al., 2018; Sisterson et al., 2019) or unravel the neural network involved in specific tasks, including sensory responses, motor activities or intellectual or emotional processes, to implement efficient Brain Machine Interfaces (BMIs) (Vansteensel et al., 2016; Wagner et al., 2018). Neural recording systems based on CMOS technology, in combination with micro-electrode arrays, can achieve very high temporal and spatial resolution and have been proved useful for assessing connectivity at the extracellular single-unit level. The suitability of these devices ultimately depends on the amount and quality of the information which can be extracted from the brain tissue and, accordingly, it is crucial to increase the number of neural signals which can be accurately and simultaneously recorded *in vivo*. In fact, in the last decades, and similar to the well-known Moore's law for transistor count scaling in dense integrated circuits (ICs), the number of single neuron cells which can be monitored using recording interfaces, either based on intracortical probes or surface sub-dural microelectrode arrays, has increased over the years. This is illustrated in the plot of Figure 1, derived from the dataset available in Stevenson (2020). Only intracortical systems published along the last three decades, i.e., approximately since the inception of the first silicon-based structures for neural recording, have been considered. The plot shows that the number of neurons which can be simultaneously recorded has increased exponentially with the year of publication, doubling approximately every 4.65±0.25 years. Anyhow, future forecasts based on this growth trend are questionable, because of fundamental limits in the size and density of microelectrodes, which cannot be decreased arbitrarily without degrading the Signal-to-Noise Ratio (SNR) of the recorded signals (Camunas-Mesa and Quiroga, 2013), or because of the induced displacement in the neural tissue which at last instance may hamper the network connectivity which is aimed to discover.

In the works represented in Figure 1, intracortical electrodes are either arranged in parallel microwire bundles (e.g., Obaid et al., 2020), or use micromachined silicon arrays (e.g., Bartolo et al., 2020), or are integrated in planar silicon-based neural probes (e.g., Mora Lopez et al., 2017), or are allocated in flexible polymer-based substrates (e.g., Musk and Neuralink, 2019). Szostak et al. (2017) gives an in-depth review of these techniques. In many cases displayed in Figure 1, multiple probes, each with multiple shanks have been used for increasing the number of recording sites. Thus, for instance, in one of the selected works, (Berényi et al., 2014), two silicon-based probes, each with 8 shanks of 32-channels, were used for a total of 512 electrodes. Similarly, in Rajangam et al. (2016), six multielectrode arrays with 96 microwires each were used totalling 576 recording sites. More recently, the trend is toward including more electrodes per individual shank or microwire bundle and, thus, for instance, the Neuropixel probe (Jun et al., 2017) has 960 sites on a single, 10 mm long, non-tapered shank with 70 × 20 μm cross-section and the Argo system (Sahasrabuddhe et al., 2020) uses a single microwire bundle with 1300 electrodes (10 mm array diameter, 18 μm wire diameter, 200 μm spacing, 1 mm length), which can be extended to 30,000 channels for surface LFP recordings.

Besides the huge amount of data to be processed and transferred, which poses significant challenges in the digital back-end of neural recording systems (Park et al., 2018a), an important bottleneck for implementing high channel count microelectrode arrays stems from the design of the active readout circuitry, which is the focus of this survey. In most of the cases represented in Figure 1, intracortical electrodes are passive, i.e., they are made up of recording sites and interconnecting wires, while the main circuitry for the acquisition, conditioning and processing of neural signals, often from multiple probes, is housed in bulky headstages (Shobe et al., 2015; Rajangam et al., 2016). Only recently, seeking to increase the number of recording sites which can addressed from the headstage, some silicon-based intracortical shanks also include active devices such as switches or small amplifiers, as in Raducanu et al. (2017). In order to reduce the form factor of headstages, specific integrated solutions, which almost invariably relies on the use of CMOS technologies, should be used for the implementation of the readout electronics. In this paper, the circuit used for recording the neural signal captured from each individual electrode will be denoted indistinctly as recording channel or neural recording Analog Front-End (AFE), and comprisees all the circuit elements from the input Low-Noise Amplifier (LNA) to the Analog-to-Digital Conversion stage (ADC) (both inclusive). Clearly, in high channel count neural recording systems, the occupation area of individual AFEs should be made as small as possible and, in fact, the density of recording channels has risen from some 5–6 AFEs/mm^2^ to more than 80 AFEs/mm^2^ in the current state-of-the-art, whilst still satisfying demanding specifications on low noise, high input impedance or low power consumption. The use of Time-Division Multiplexing (TDM) techniques, in which occupation area is traded-off with operation frequency, have played a prominent role on this accomplishment.

TDM makes it possible to totally or partially share AFE elements at different time slots between different electrodes. Compared to other multiplexing techniques such as frequency-division multiplexing (Mikawa et al., 2020), TDM does not suffer from signal overlapping issues in frequency domain, and allows to reusing circuit blocks without penalizing to first order power consumption. This is because although shared elements have to increase their power consumption proportionally to the higher bandwidth requirements in order to preserve the same neural recording sampling rate, such increment is essentially compensated by the fact that only one element is used instead of multiple slower elements. Another advantage of TDM is the improved tolerance against mismatch between recording channels as a single element is shared between different electrodes, thus reducing discrepancies between the recorded neural signals. Obviously, the more AFE elements are shared by means of multiplexing, the higher the area reduction which can be attained for the complete readout circuitry. In a limit case, the largest area saving could be accomplished if a single AFE is multiplexed between different electrodes (Jun et al., 2017; Raducanu et al., 2017; Sharma et al., 2018), however, as will be shown in this paper, this raises undesired effects which should be tackled.

This work aims to review the implementation strategies and restrictions for time multiplexing neural recording AFEs, and analyses the main advantages and drawbacks of the proposed techniques and architectures. The paper is organized as follows. Section 2 details the main concerns about the AFE-electrode interface. Section 3 introduces AFEs for neural recording applications. Section 4 describes the basics of TDM and section 5 presents a classification of the reported neural recording architectures depending on the position of the analog multiplexer in their signal paths. Section 6 describes the main architectures for TDM at the AFE input, together with their advantages and drawbacks. Finally, section 7 offers some conclusions and suggestions for future research.

## 2. Electrode-AFE Interface

AFEs in multi-channel systems are usually placed relatively far from the recording electrodes. The interconnection wires between the electrode array and the AFEs severely limit the electrode density and reduce the efficiency of the neural probe's occupation area. However, some silicon-based devices allow the integration of one or more AFE stages along with the electrodes, by splitting the AFE into two parts: one placed on the headstage and the other on the probe shank(s). The most employed stage to be integrated next to the electrodes is the input amplifier (IA). The main advantages and drawbacks of employing or not this active circuitry along with the electrodes can be disclosed in their impact on electrode crosstalk and its noise contribution to the system. The considerations presented throughout this section will apply for recording both local field potentials (LFPs), which are signals comprising the combination of synaptic and network activities within a local brain region with an oscillation frequency from 0.5 to 500 Hz (Muller et al., 2015), and action potentials (APs), which are rapid rises and subsequent falls in voltage or membrane potential across a cellular membrane in a frequency band from 0.25 to 10 kHz (Muller et al., 2015).

### 2.1. Crosstalk in Electrode-AFE Interfaces

Electrical crosstalk is one of the most significant scaling limitations of multi-channel recording devices. For neural applications, the crosstalk level has to be below 1% of the recorded signal level to make it negligible compared with the background noise (Najafi et al., 1990). This crosstalk can be classified according to where it occurs: electrode crosstalk defines the crosstalk from the electrodes to the AFE, i.e., from the shank to the base of the device; crosstalk takes into account the impact on the multiplexer output of non-activated channels. This last type of crosstalk will be analyzed in section 4.

**Figure 2:**
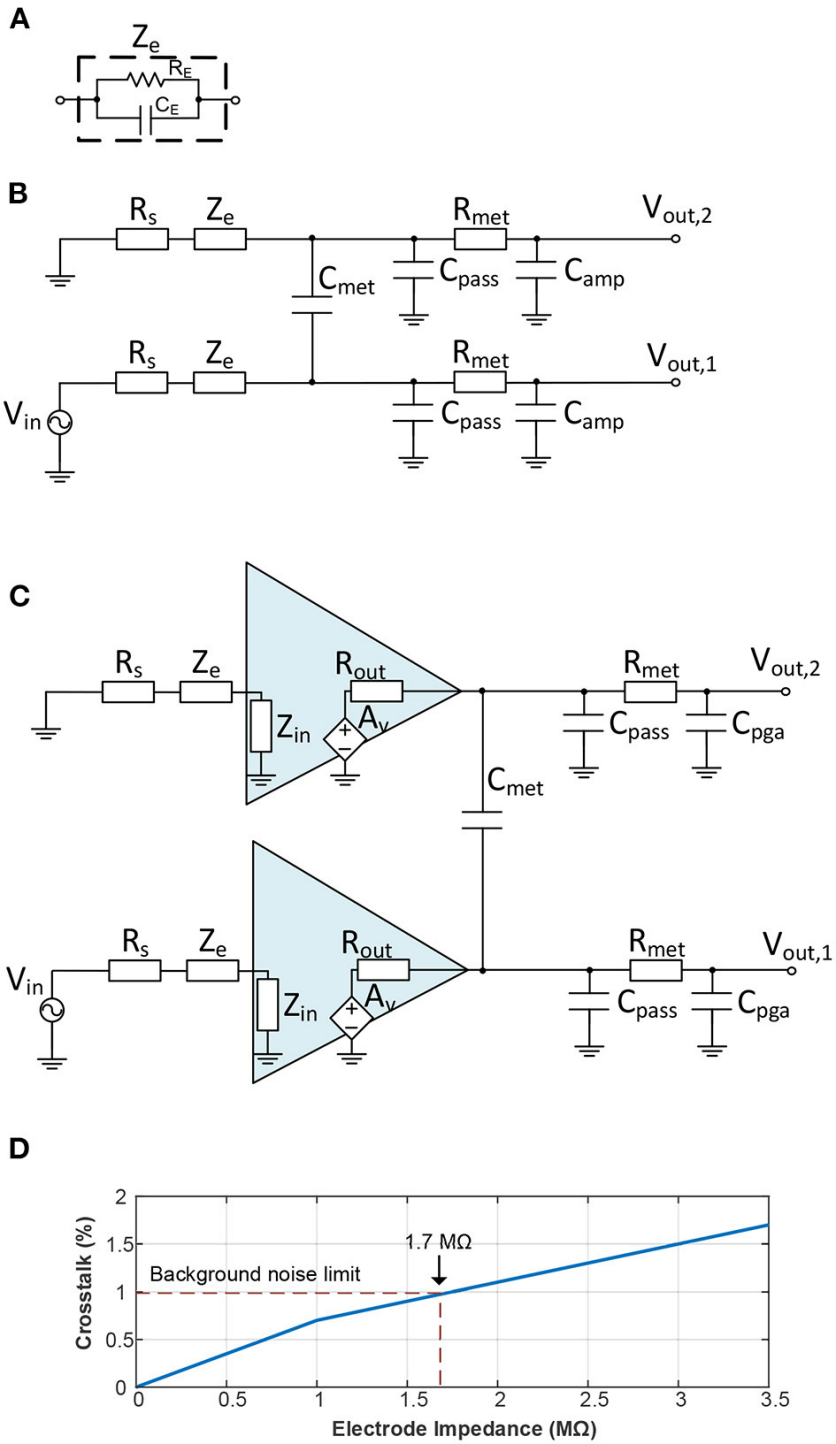
Crosstalk models for electrode-AFE interface. **(A)** Equivalent electrode impedance model. **(B)** Equivalent circuit model for electrode crosstalk without active electrode-AFE interface. **(C)** Equivalent circuit model for electrode crosstalk with active electrode-AFE interface. **(D)** Crosstalk at the electrode-AFE interface against the electrode impedance (Du et al., 2009).

In high-channel-count devices, the space between adjacent electrodes and between interconnection wires is largely reduced while the dielectric layers below and above the electrodes remain constant. The coupling capacitance between electrodes thus increases because of the reduced space, thereby increasing the electrical crosstalk. A simplified scheme which models the crosstalk from the shank to the AFE was proposed in Najafi et al. (1990) and further developed in Du et al. (2009) and Seidl et al. (2012) (see Figure 2B). It should be noted that (Seidl et al., 2012) also demonstrated that the switches placed along with the electrodes have a negligible effect on crosstalk, so they were not included in the model. For the AFE to be integrated within the neural probe, the capacitive coupling between metal lines in the external wires (Du et al., 2009) was also excluded. To further develop this approach, Figure 2C shows a model which also includes an amplifier adjacent to the electrodes, similar to Lopez et al. (2014). The circuit elements with their corresponding values (taken from Du et al., 2009; Seidl et al., 2012) are described as follows:

- *R*_*s*_ is the spreading resistance encountered by the current propagating out into the fluid near to the electrode. It has been reported to be about 10 kΩ (Du et al., 2009).
- *Z*_*e*_ represents the equivalent impedance of the electrode-electrolyte interface (from now on simplified as electrode impedance) which is modeled by a resistive *R*_*E*_ and a capacitive *C*_*E*_ component, see Figure 2A (Du et al., 2009). This impedance is, then, a frequency-dependent parameter. For this analysis, a 20 μm diameter Pt electrode has been taken with an impedance measured at 1 kHz of about 1.2 MΩ (Seidl et al., 2012).
- *C*_*met*_ describes the capacitive coupling between adjacent lines. It was estimated as 0.1 pF (Du et al., 2009).
- *C*_*pass*_ is the estimated capacitance of the metal lines with the extracellular fluid. This value was set as 2.7 pF (Du et al., 2009).
- *R*_*met*_ represents the equivalent resistance between the metal traces to the input of the amplifier (programmable-gain amplifier, PGA, in the case of the active electrode-AFE interface). Its value was about 500 Ω (Du et al., 2009).
- *C*_*amp*_ (only for non-active electrode-AFE interfaces) models the input capacitance of the AFE and was set at about 12 pF (Du et al., 2009).
- *C*_*pga*_ (only for active electrode-AFE interfaces) represents the input capacitance of the PGA and it is about 12 pF.
- *Z*_*in*_ (only for active electrode-AFE interfaces) is the input impedance of the amplifier next to the electrode. It is above 70 MΩ.
- *R*_*out*_ (only for active electrode-AFE interfaces) describes the output resistance of the amplifier next to the electrode. This value is about 50 kΩ.

Simulations carried out in the SPICE software environment in Du et al. (2009), have demonstrated the influence of the electrode impedance in the crosstalk between channels. Electrode impedances larger than 1.7 MΩ produce crosstalks between channels above the 1% (Du et al., 2009), significantly reducing the SNR. This is illustrated in Figure 2D, which replicates the analysis provided in Du et al. (2009). It is worth observing that these results also included the influence of the capacitive coupling of the metal lines for the connection with an external AFE, which is not the case of placing the AFE at the base of the probe (Du et al., 2009).

On the other hand, placing the amplifier adjacent to the electrodes isolates the impedance of the electrodes from the interconnection wires (Lopez et al., 2014). This makes the crosstalk dependent on the output resistance of the amplifier due to the fact that this resistance is in this model the equivalent input impedance seen from the interconnection wires (Lopez et al., 2014). Thus, crosstalk results are largely improved (Lopez et al., 2014). For instance, crosstalk values below 0.1% have been reported by including amplifiers along with the electrodes (Mora Lopez et al., 2017).

### 2.2. Noise in Electrode-AFE Interfaces

**Figure 3:**
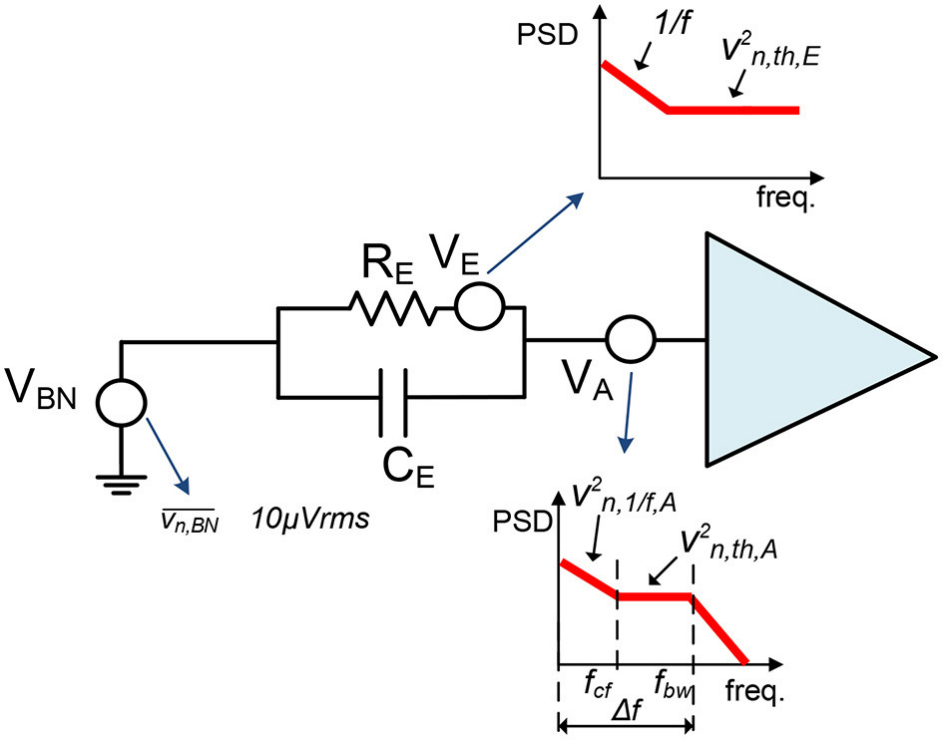
Simplified scheme of the three main noise sources at the Electrode-AFE interface: the biological or background noise (VBN), the electrode noise (VE), and the noise from the recording electronics (VA).

One of the most significant aspects of neural recording devices is how different noise sources degrade the signal of interest. At the electrode-AFE interface, three main noise sources can be distinguished: biological or background noise, electrode-electrolyte interface noise and the noise from the recording electronics (Obien et al., 2015). This is illustrated in Figure 3 (Valtierra et al., 2020).

The background noise, *V*_*BN*_ in Figure 3, comprises the electrical activity of other cells surrounding the recording electrode (Obien et al., 2015). This noise source is usually quoted as $\bar{v_{n,BN}}\approx 10\mu V_{rms}$ although its spectral density distribution is not generally defined (Chandrakumar and Markovic, 2017; Valtierra et al., 2020).

The electrode impedance also adds noise to the signal chain, *V*_*E*_ in Figure 3. The power spectral density (PSD) function of this noise source at low frequencies (below 10 Hz) is proportional to 1/f (Obien et al., 2015). Above these frequencies, the thermal noise becomes the main noise contributor and its value is given by (Obien et al., 2015):

(1) $$\begin{array}{l} \bar{v_{n,E}^{2}}=4\cdot k\cdot T\cdot Re\left(Z_{e}\right)\cdot \Delta f \end{array}$$

where *k* is the Boltzamnn constant, *T* is the temperature, *Re*(*Z*_*e*_) is the real part of the electrode impedance and Δ*f* the recording bandwidth. The resulting PSD is simplified in Figure 3.

Finally, the recording electronics, i.e., the AFE, also introduce noise to the signal of interest. The main noise contributor of the AFE is generally the IA because it involves the first amplification stage. The IA's PSD is conventionally divided into three sections: (i) from low frequencies to the corner frequency, *f*_*cf*_, the flicker noise contribution, *v*_*n*, 1/*f, A*_, dominates the noise PSD; (ii) from the corner frequency to the amplifier frequency bandwidth, *f*_*bw*_, the main noise contributor is the thermal noise, *v*_*n, th, A*_; and (iii) above this frequency the noise is filtered and can be neglected (Razavi, 2001) (Figure 3). Both flicker and thermal noise contributions will depend on the amplifier topology and operation region. In biomedical applications, amplifiers are commonly biased in sub-threshold region (Muller et al., 2015; Delgado-Restituto et al., 2017; Valtierra et al., 2020). Hence, for an IA employing an operational-transconductance amplifier (OTA), the thermal and flicker noise contributions can be respectively estimated by (Valtierra et al., 2020):

(2) $$\begin{array}{l} \bar{v_{n,th,A}^{2}}=\frac{4\cdot k\cdot T\cdot \gamma \cdot \eta \cdot V_{t}}{I_{b}}\Delta f \end{array}$$

(3) $$\begin{array}{l} \bar{v_{n,1/f,A}^{2}}=\frac{K_{F}}{W\cdot L\cdot C_{ox}\cdot f}\Delta f \end{array}$$

where *V*_*t*_ is the thermal voltage, η the sub-threshold slope, *gamma* = 1/2 for the sub-threshold region, *I*_*b*_ the current through the OTA, *C*_*ox*_ is the gate-oxide capacitance, *K*_*F*_ is a flicker parameter dependent on the specific fabrication process and *W* and *L* are the width and length of the OTA.

Integrating this IA adjacent to the electrodes makes the power and area constraints of this stage even more restricted. In terms of the power consumption, neural devices in contact with the tissue have to be designed within the allowed limit of < 1°C of brain tissue heating (Kim et al., 2007). Furthermore, the active area of the shank has to be minimized to increase the number of readout channels (Mora Lopez et al., 2017). Therefore, it can be observed from equations 2 and 3 that the thermal and the flicker noise contribution of the amplifier can be penalized.

In terms of thermal noise, the power consumption of the amplifiers located adjacent to the electrodes increases the shank heating, so the current through these amplifiers, *I*_*b*_, has to be minimized. From equation 2 can be noted that the thermal noise contribution of the active electrode-AFE interfaces would theoretically be larger than in passive shanks. Nevertheless, as demonstrated by the finite element method simulations carried out in Mora Lopez et al. (2017), this power limitation depends on the structure employed for the probe. Thus, up to 20 mW of power dissipation in the shank can be tolerated without increasing the temperature of the tissue by one degree (Mora Lopez et al., 2017). This keeps the amplifier's power consumption and, consequently, the thermal noise contribution of this stage, at the same level as in conventional AFE structures by properly designing the probe.

In terms of the occupation area, reducing the active area located along with the electrodes makes it possible to increase the number of recording channels and, in turn, the recording density of the neural interface. Keeping the electrode area constant, the area will increase with the size of the amplifier located adjacent to the electrodes, establishing a trade-off between the amplifier's occupation area and the amplifier's flicker noise contribution (see Equation 3). While no such huge impact has been reported in the APs band, the effect of this noise becomes significant for LFP recording. This has been assessed by employing the integrated input-referred noise (IRN), which is the total integrated noise over the band of interest referred to the input of the circuit (Razavi, 2001). This is a widely used measure to evaluate the noise performance of the circuits. In this case, the IRN for the system presented in Mora Lopez et al. (2017) in the AP band is about 6.36 μ*V*_*rms*_ while the IRN in the LFP band reaches 10.32 μ*V*_*rms*_. Therefore, the amplifier adjacent to the electrodes has to be carefully designed in terms of occupation area to not penalize the IRN of the circuit, specially at low frequencies.

## 3. Neural Recording AFEs

**Figure 4:**
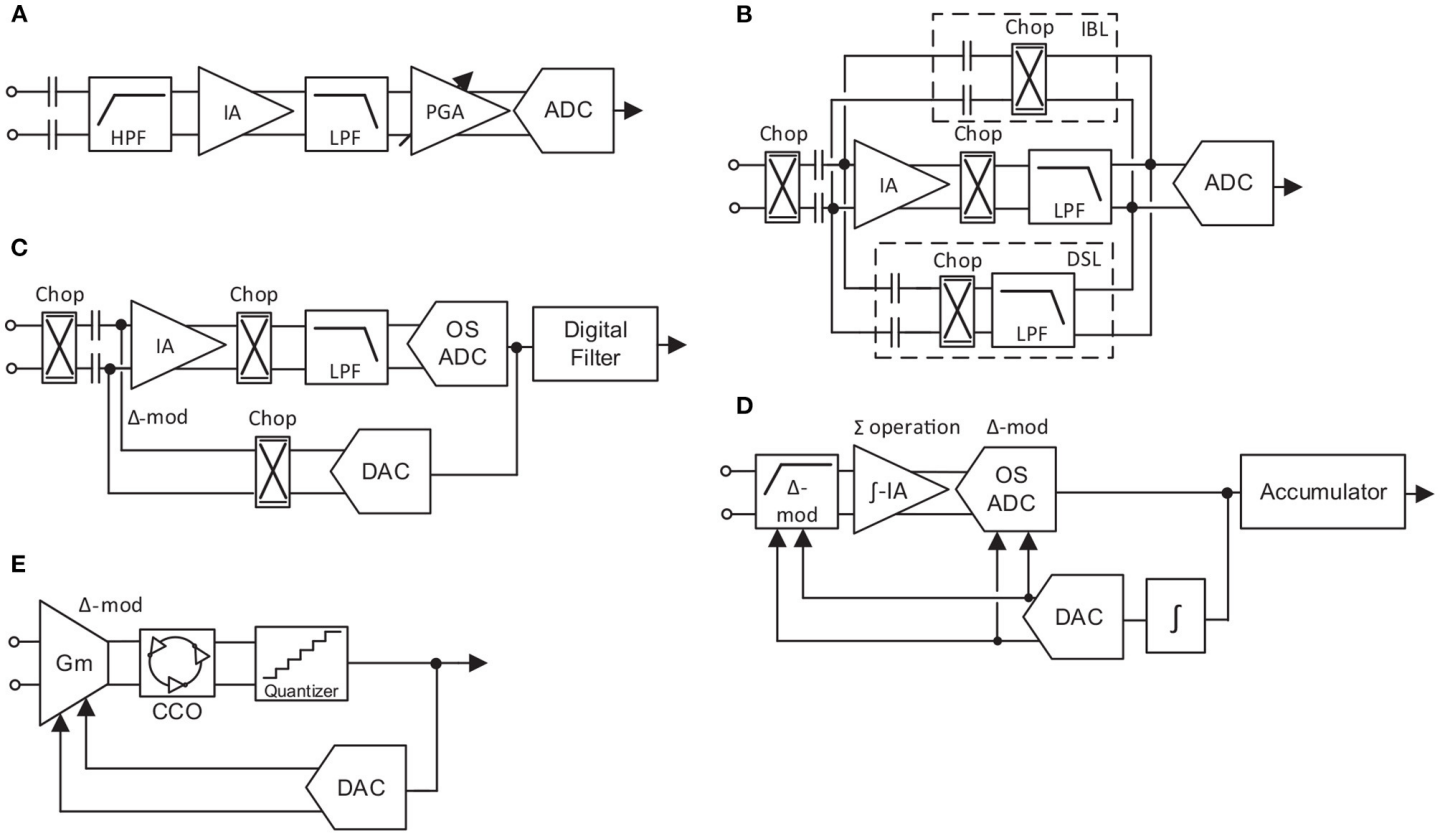
Block diagram of high-performance neural AFEs. **(A)** Continuous-Time AFE topology. **(B)** Chopper-stabilized AFE topology. **(C)** Chopper-based Δ-AFE topology. **(D)** Δ^2^Σ AFE topology. **(E)** VCO-Based ΣΔ AFE topology.

Neural recording AFEs are traditionally made up of an LNA, a PGA, an anti-aliasing filter and an ADC. However, over the years this standard has gradually been adapted according to the requirements of each specific system and with the purpose of optimizing the performance of the circuit. Thus, five different high-performance approaches for neural AFEs have been simplified and illustrated in Figure 4.

The conventionally employed AC-coupled topology, continuous-time (CT) AFE (Brenna et al., 2016; Delgado-Restituto et al., 2017; Park et al., 2018a,b), is shown in Figure 4A. The basic structure of the LNA is presented in Harrison and Charles (2003). This topology obtains a high input impedance, which is desired to be as high as possible to reduce the attenuation of the signal due to the electrode impedance (Pazhouhandeh et al., 2020b), by reducing the size of the input capacitors. Moreover, the high-pass filter required to reject the input DC offset from electrodes is implemented in the IA, where the pole of the IA transfer function is set by the input capacitors and a pair of feedback resistors. Using pseudoresistors is a common technique for setting this pole at sub-Hz frequencies without penalizing the area of the AFE. However, these resistors conventionally present large temperature and process variations (Sharma et al., 2021). Furthermore, due to the lack of specific techniques for low-frequency noise reduction, IAs are usually made up of large input transistor area. Another significant disadvantage relies on the prone to saturation of the IA to input artifacts due to its high gain and its large time constant.

The chopper stabilization technique is a widely employed method to reduce the low frequency noise components of an amplifier by splitting, in the frequency domain, the flicker noise components from the signal of interest (Enz and Temes, 1996). In recent years, DC-coupled chopper-based AFE topologies (Figures 4B,C) have proven their efficiency in further reducing the flicker noise component of the IA. In these architectures, the input impedance is inversely proportional to the chopping frequency and the input capacitor value. Herein, two main impedance boosting techniques have been reported: implementing an impedance boosting loop (IBL) by means of a positive feedback network (Fan et al., 2011) (Figure 4B), or/and employing an auxiliary input path (Chandrakumar and Markovic, 2017) (not shown in Figure 4B), penalizing the IRN of the system. These DC-coupled topologies also require a mechanism to remove the input DC offset from the electrodes. One widely adopted solution consists of employing a DC SERVO LOOP (DSL) (Figure 4B) in the analog domain (Fan et al., 2011; Chandrakumar and Markovic, 2017; Lee and Song, 2019; Samiei and Hashemi, 2019) or in the digital domain (Muller et al., 2015) (not illustrated). Another approach is based on employing the Δ-modulation technique. This technique relies on tracking differences between successive samples which inherently implements a high-pass filter. In this way, the applied technique consists of working with Δ-signals by feeding the previous (Johnson et al., 2017) or the predicted (Kim et al., 2018) value of the signal (Figure 4C) into the input of the IA by a mixed-signal loop. This method usually increases the dynamic range (DR) of the AFE at the cost of requiring an oversampled ADC (OS ADC).

Besides conventional AFE topologies, some alternatives based on Δ Σ schemes have been presented (Kassiri et al., 2017; Pazhouhandeh et al., 2020a). In contrast with the Δ-modulation technique, the Σ operation relies on the integration of the signal through the summation of successive samples (Carusone et al., 2011). A similar approach of continuous-time Δ Σ AFE is reported in Chandrakumar and Markovic (2018). Some of these architectures are also known as ADC-direct schemes and do not have IAs. Promising architectures based on this technique relies on applying twice the Δ-modulation and are know as Δ^2^ Σ AFEs (Figure 4D) (Pazhouhandeh et al., 2020a). In these systems, the signal is Δ-modulated at the input, similar to Figure 4C). Then, the signal is integrated by the Σ operation carried out during the amplification stage and, finally, Δ-modulated again in the analog-to-digital conversion. Thus, the SNR is largely increased (Pazhouhandeh et al., 2020a). As in the chopper-based AFEs, however, the input impedance depends on the modulation frequency. To improve that, a Δ-modulation opamp-less topology was presented in Pazhouhandeh et al. (2018, 2020b) which increases the input impedance to the GΩs.

Finally, AFEs that rely on a conversion of the signal amplitude to the frequency domain, time/frequency based AFEs, show large efficiency in terms of occupation area (Tu et al., 2017; Jeon et al., 2019). Herein, voltage-controlled oscillators (VCOs) based circuits, which transforms the input signal amplitude into different oscillation frequencies, have recently proven to be an efficient low-power alternative to conventional AFEs (Jiang et al., 2017) and low-frequency filters (Leene and Constandinou, 2017). In these topologies, an AC-coupled input transconductance, *G*_*m*_, converts the input voltage to current, which is translated to phase by a current-controlled oscillator (CCO) and, finally, converted to the digital domain by a quantizer. Due to the open-loop nature of the AFE, for large input signals the *G*_*m*_ suffers from strong non-linearity, requiring an extra digital circuit calibration at the output of the quantizer. A different approach to implementing VCO-based AFEs is reported in Prabha et al. (2015); Tu et al. (2017); Jeon et al. (2019) and shown in Figure 4E. To solve the dynamic range problem, a mixed-signal loop is employed to perform a ΔΣ operation. As in the previously presented topologies, these Δ-signals at the input eliminate the DC-offset from the electrodes and allow the *G*_*m*_ to work in the linear region for a larger input range. However, the low-frequency noise contribution of the *G*_*m*_ is not reduced and large input transistors are needed to keep it within the system's noise margins.

### 3.1. Comparison of State-of-the-Art

**Figure 5:**
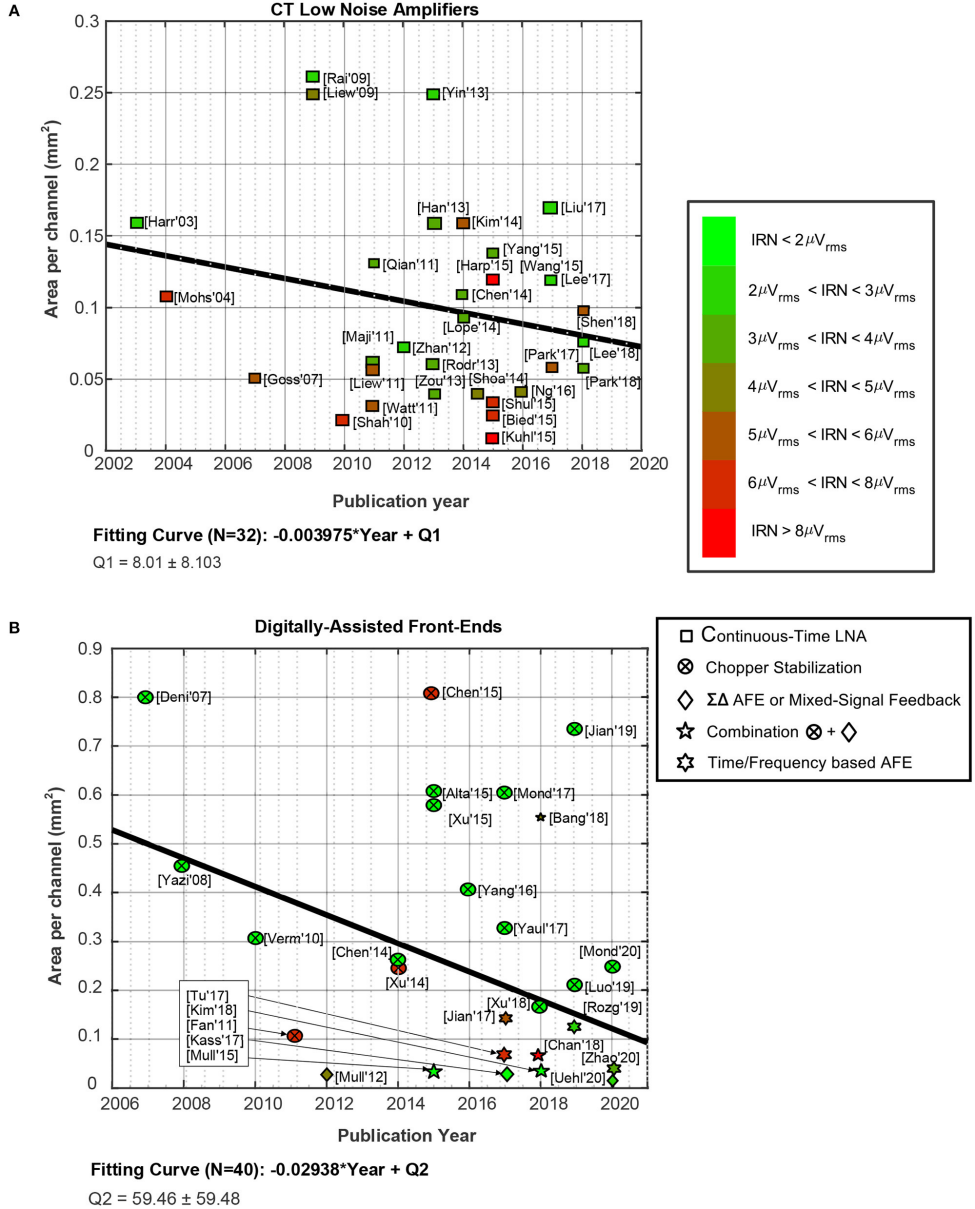
Evolution of the form factor of neural AFEs over the years. The green-red scale indicates the value of the IRN of each reported circuit. Fitting curves represent the trend over the years of the area per channel. **(A)** For CT LNAs. **(B)** For digitally-assisted AFEs.

**Figure 6:**
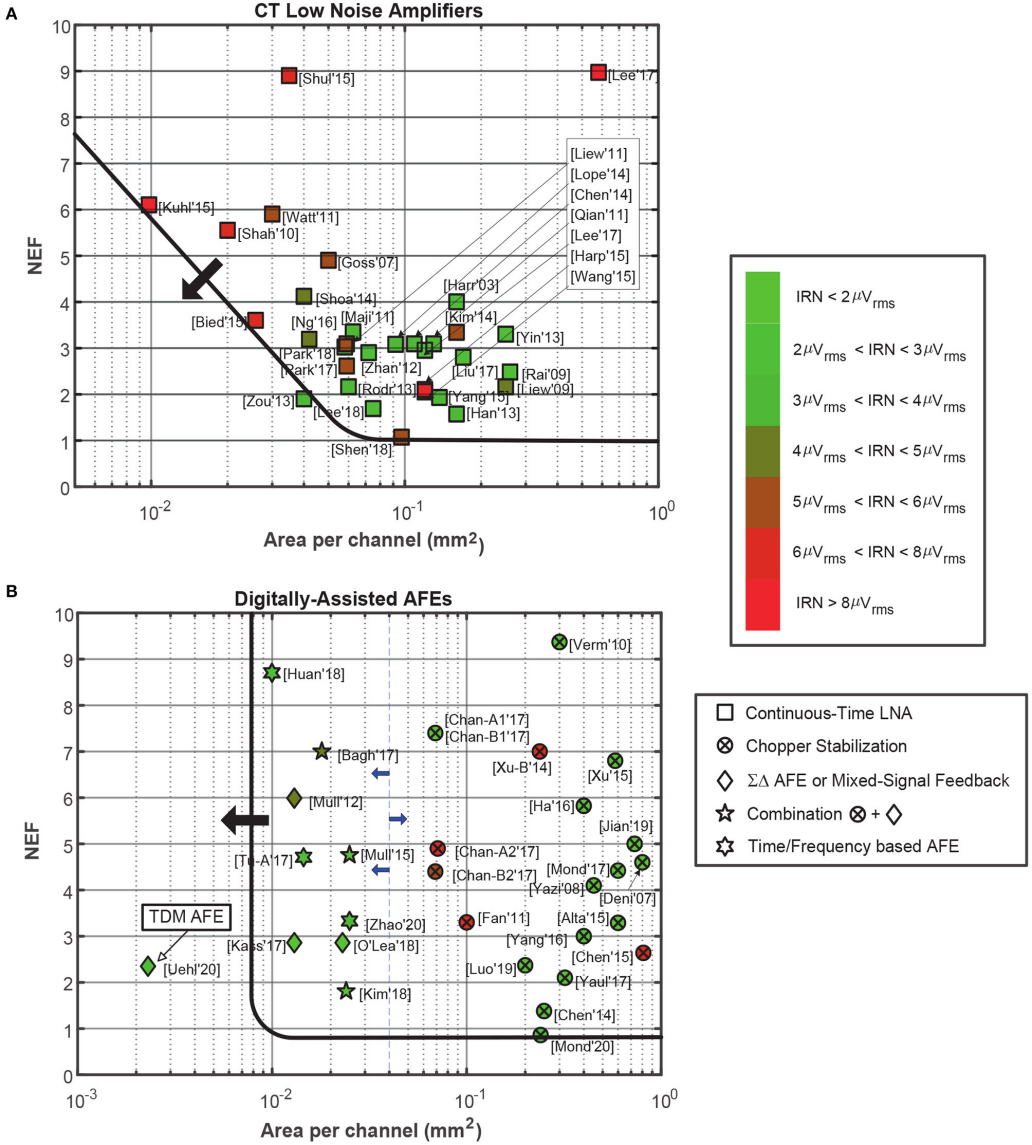
Comparison of state-of-the-art LNAs and AFEs topologies in terms of NEF and area per channel. The green-red scale indicates the value of the IRN for each reported circuit. In both figures, arrows indicate the limit on the trend of current circuits, and how future approaches should be beyond the marked line. **(A)** For CT LNAs. **(B)** For Digitally-Assisted AFEs.

**Figure 7:**
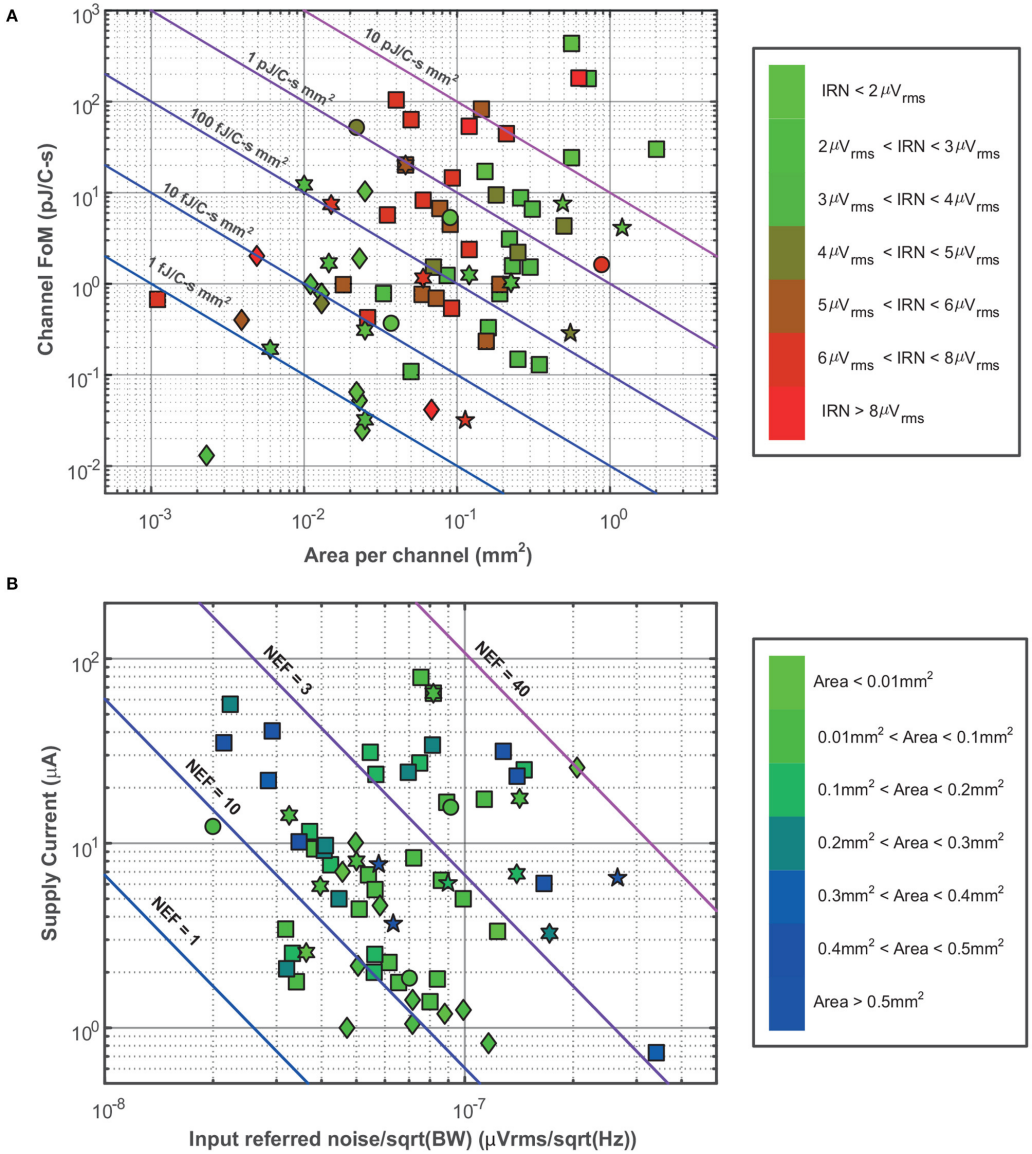
Comparison of state-of-the-art LNAs and AFEs topologies in terms of Channel FoM and normalized IRN. **(A)** Channel FoM vs. area per channel comparison. The green-red scale indicates the value of the IRN of each reported circuit. The lines shows different FoM per area values as references. **(B)** Supply current vs. normalized IRN comparison. The green-blue scale indicates the value of the occupation area of each reported circuit. The lines show different NEF values as references. The employed symbol code is the same as shown in Figure 6.

A comparison of state-of-the-art AFEs and LNAs topologies are illustrated in Figures 5–**7**. It is worth observing that a green-red scale is provided in the figures to evaluate the IRN of each work: the green represents the lowest IRN values and the red the highest IRN values.

Firstly, Figure 5 represents the evolution of the form factor over the last years for CT LNAs (Figure 5A) and for digitally-assisted AFEs (Figure 5B). It should be noted that digitally assisted AFEs comprise all AFE architectures presented in section 3, except for CT AFEs. The occupation area saving over the years for CT LNAs (Figure 5A) has not been as high as in the case of digitally-assisted AFEs (Figure 5B). This is mainly due to the fact that first topologies involving chopper-based amplifiers used to have large input capacitors to improve the IRN results.

On the other hand, Figure 6 illustrates the noise efficiency factor (NEF), parameter which represents the performance of a circuit in terms of its noise contribution and power consumption, against the area per channel. The NEF is defined as:

(4) $$\begin{array}{l} NEF=v_{ni,rms}\cdot \sqrt{\frac{2\cdot I_{t}}{V_{t}\cdot 4\cdot k\cdot T\cdot \Delta f\cdot \pi }} \end{array}$$

where *v*_*ni, rms*_ the IRN of the amplifier and *I*_*t*_ the total current through the circuit. This parameter is widely used to illustrate the performance of neural AFEs. Thus, while in Figure 6A, this comparison is made for CT LNAs, in Figure 6B, this comparison is made digitally-assisted AFEs. Note how the NEF largely depends on the area of the LNA due to the impact of the flicker noise contribution in the case of the CT LNAs (Figure 6A). In Figure 6B, it can be observed how chopper-stabilized AFEs offer the lowest IRN at the cost of increasing the area per channel. Proposed solutions based on Δ Σ AFEs and mixed-signal feedback offer some of the best performances in terms of noise and area per channel.

Finally, Figure 7A compares the channel figure-of-merit (FoM) against the area per channel for different AFE architectures. This FoM represents the performance of the circuit in terms of power, resolution and bandwidth and is given by:

(5) $$\begin{array}{l} Channel\;FoM\left(DR\right)=\frac{P_{ch}}{2BW\cdot 2^{ENOB\left(DR\right)}} \end{array}$$

where *P*_*ch*_ is the power consumption per channel and *ENOB*(*DR*) = (*DR*(dB)- 1.76)/6.02, represents the equivalent number of bits for the DR of the system. Figure 7B compares the LNA supply current with the normalized IRN, which is the result of multiplying the IRN by $\sqrt{BW}$. In both comparisons, CT LNAs show better results than digitally-assisted AFEs, due to the employment of low-power analog blocks instead of complex mixed-signal loops. Moreover, CT LNAs also usually have lower bandwidths than digitally-assisted AFEs, especially those using the chopper-stabilization technique. In Figure 7B, the IRN scale has been replaced by a red-blue scale which represents the occupation area per channel.

## 4. Time-Division Multiplexing

TDM is a technique widely employed in communication systems. It relies on dividing the data from *M*-channels into *M* different time slots of the same output signal. Thus, after multiplexing, the signal from the *M* channels is shared by the same AFE block/s, reducing the number of instances of each multiplexed block employed by *M* − 1. The main advantage of the TDM technique in neural recording AFEs therefore lies in area saving, which will scale up with the number of multiplexed stages. The technique is carried out by an analog multiplexer, the operating frequency of which, *f*_*mux*_, has to be at least 2 · *M*-times faster than the signal bandwidth, *fb*. The bandwidth of the subsequent block/s therefore has to be about *M* times larger than in non-multiplexed topologies, leaving the system's overall power consumption the same.

One of the main drawbacks of this technique is related to noise folding. Employing larger bandwidth blocks increases in-band noise, which will be folded to the baseband. Although anti-aliasing filters are used to reduce this spectral folding, if the multiplexer is located in one of the first stages of the AFE, this filter becomes harder to implement and another approach must be adopted. This problem is described in more detail in section 6.

Another noise source to take into account during system design is the crosstalk from an analog multiplexer. This crosstalk can be disclosed in four different components: (i) capacitive coupling between the input metal lines of the multiplexer; (ii) the finite off-resistance of the switches; (iii) time-adjacent channel crosstalk; (iv) capacitive coupling through the parasitic capacitance of the transistor used as a switch. The first three crosstalk sources can be ignored. In the first case, the impact of the capacitive coupling can be avoided by applying layout techniques such as the careful shielding of each input line. In the second, the subthreshold conduction of the switches is negligible due to the large back-bias effect in low-voltage topologies. The off-resistance is in the order of hundreds of GΩ, which does not represent a crosstalk source in the circuit (Seidl et al., 2012). Time-adjacent channel crosstalk reveals the multiplexer's ability to charge/discharge the load capacitors during the active period of a channel. If the multiplexer response is slow, a residual charge will appear between two time-adjacent channels, resulting in crosstalk noise. The time constant defined by the on-resistance of the switches along with the load capacitors of the circuit should therefore be designed to be as small as possible, in order to suppress this crosstalk source.

The effect of the capacitive coupling through the parasitic capacitance of the transistor can have a real impact at the multiplexer output (Chae et al., 2008). Multiplexer crosstalk can be defined as the effect of the turned-off channels at the output of the multiplexer. A complete mathematical analysis of this crosstalk effect is provided in Chae et al. (2008). The results from this analysis show that the value of output resistance of the previous stage strongly influences the crosstalk results. For instance, for a resistance value of 4 kΩ, the crosstalk noise is around −110 dB at 10 kHz (Chae et al., 2008). Therefore, by properly setting this value, this crosstalk source can be neglected.

## 5. Taxonomy of Neural Recording Multiplexing Systems

**Figure 8:**
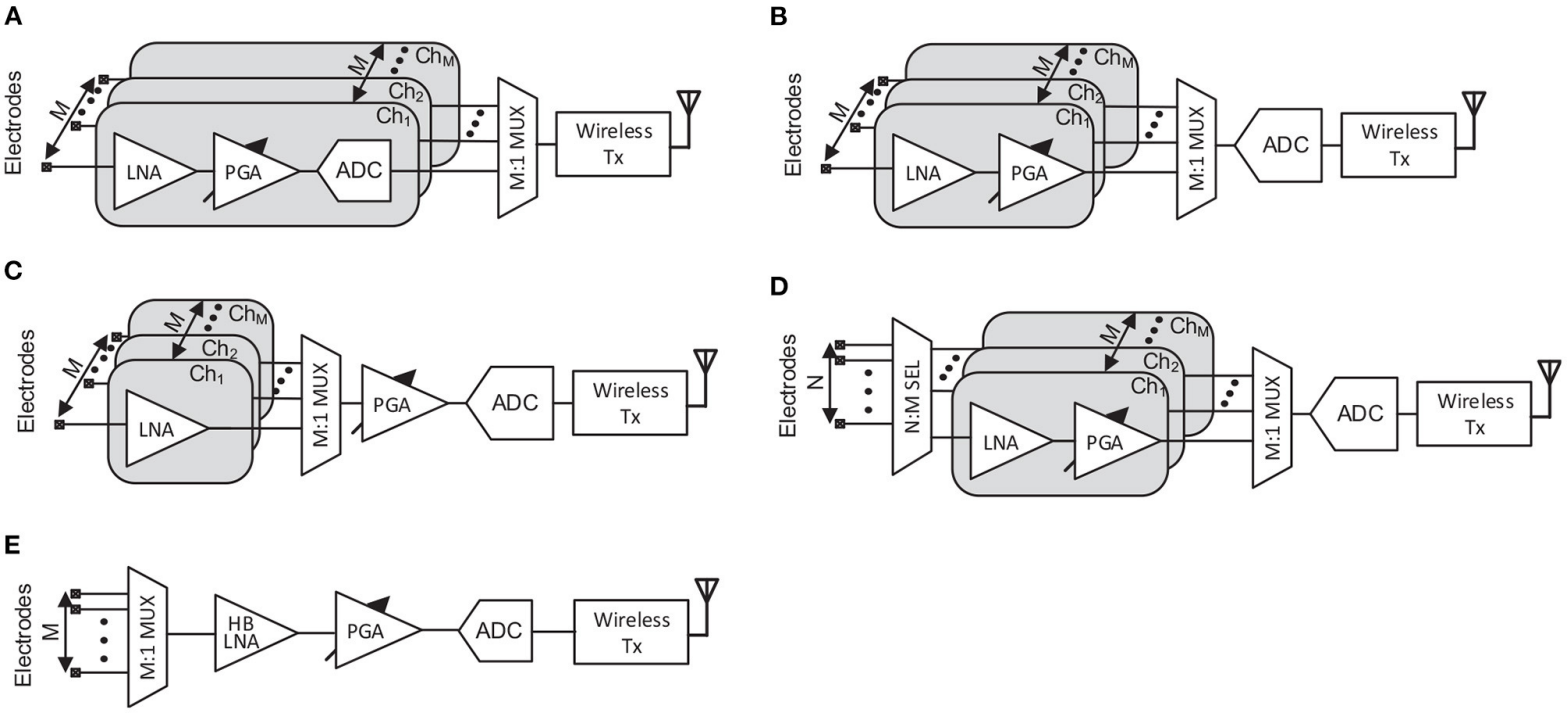
Taxonomy of different AFEs depending on the location of the multiplexer. **(A)** Non-multiplexed AFE topology. **(B)** ADC sharing AFE topology. **(C)** PGA sharing AFE topology. **(D)** Switch array AFE topology. **(E)** Time-division-multiplexing AFE topology.

Some of the main building blocks of neural recording AFEs presented in section 3 can be multiplexed to save area. Neural recording multi-channel AFE topologies can thus be classified by the position of the multiplexer in the signal path and, consequently, by the number of multiplexed blocks (Figure 8). Note that Figure 8 does not show the anti-aliasing filter. This is because although this low-pass filter is commonly embedded within the LNA, some works, such as Angotzi et al. (2018), include it in other stages.

### 5.1. Non-multiplexed AFE Topology

In non-multiplexed AFE topology (see Figure 8A), each channel is recorded by a low-rate (low sampling frequency per channel) low-power AFE. For *M* independent recording channels, *M* independent AFEs are therefore required. Herein, all the presented architectures in section 3 are suitable for this kind of topology. In terms of the electrode-AFE interface, the constraints of the active area in the shank make the integration of these AFEs along with the electrodes, beforehand, not suitable. Thus, integrating them far from the electrodes relaxes the size and power constraints of the AFE. This enables the inclusion of additional on-chip functionality for the AFE. Employing an AFE per channel also involves mismatch errors in multi-channel topologies (Ng and Xu, 2016). Low-frequency neural signals relax the bandwidth requirements of the AFE's blocks, leading to a reduction of the power consumption of each block. In addition, the design of the non-multiplexed AFE structure must meet the conventional requirements of neural recording AFEs: high input impedance, low-noise, low-power, small occupation area, large common-mode rejection ratio (CMRR), and large DC offset rejection (Muller et al., 2015; Chandrakumar and Markovic, 2017, 2018).

In terms of the ADCs used, despite using a low sampling frequency, conventionally up to few kHz, the need for one ADC per channel requires very careful design in order not to largely increase the area and power consumption of the neural recording IC. Successive-approximation (SAR) ADCs have generally shown good results for this kind of topologies (Gao et al., 2012; Brenna et al., 2016; Delgado-Restituto et al., 2017; Johnson et al., 2017), specially for providing low power consumption for resolutions about 8 to 10 bits (Delgado-Restituto et al., 2016). After conversion, the signal is multiplexed, typically by employing data serializers (Park et al., 2018a,b) (Figure 8A). In the digital domain, the signal presents higher noise margins and is more stable against crosstalk and other noise sources than in the analog domain, making it more suitable for multiplexing.

### 5.2. ADC Sharing and PGA Sharing AFE Topologies

One of the most popular approaches for multi-channel architectures is to use a single ADC shared by all channels (Figure 8B). Theoretically, this reduces the form factor and the power consumption of the IC. This topology is based on *N* structures with *M* channels per structure sharing a single ADC (*N* ADCs for the whole system) (Wattanapanitch and Sarpeshkar, 2011; Zou et al., 2013; Bagheri et al., 2014; Yeager et al., 2014; Liu et al., 2017). This approach has the same problems and advantages as the non-multiplexed AFE topology in terms of the AFE-electrode interface. Regarding the electrical properties of the AFE itself, after amplification the signal is directed toward the ADC by means of TDM. The *M* times increased sampling frequency increases the power consumption of the ADC and could even require driving buffers at the input of the converter (Noshahr et al., 2020). Most of the topologies presented in section 3 are suitable for being multiplexed at the ADC stage. However, those with a digitally-assisted loop will require memory blocks to store the information for each channel.

**Figure 9:**
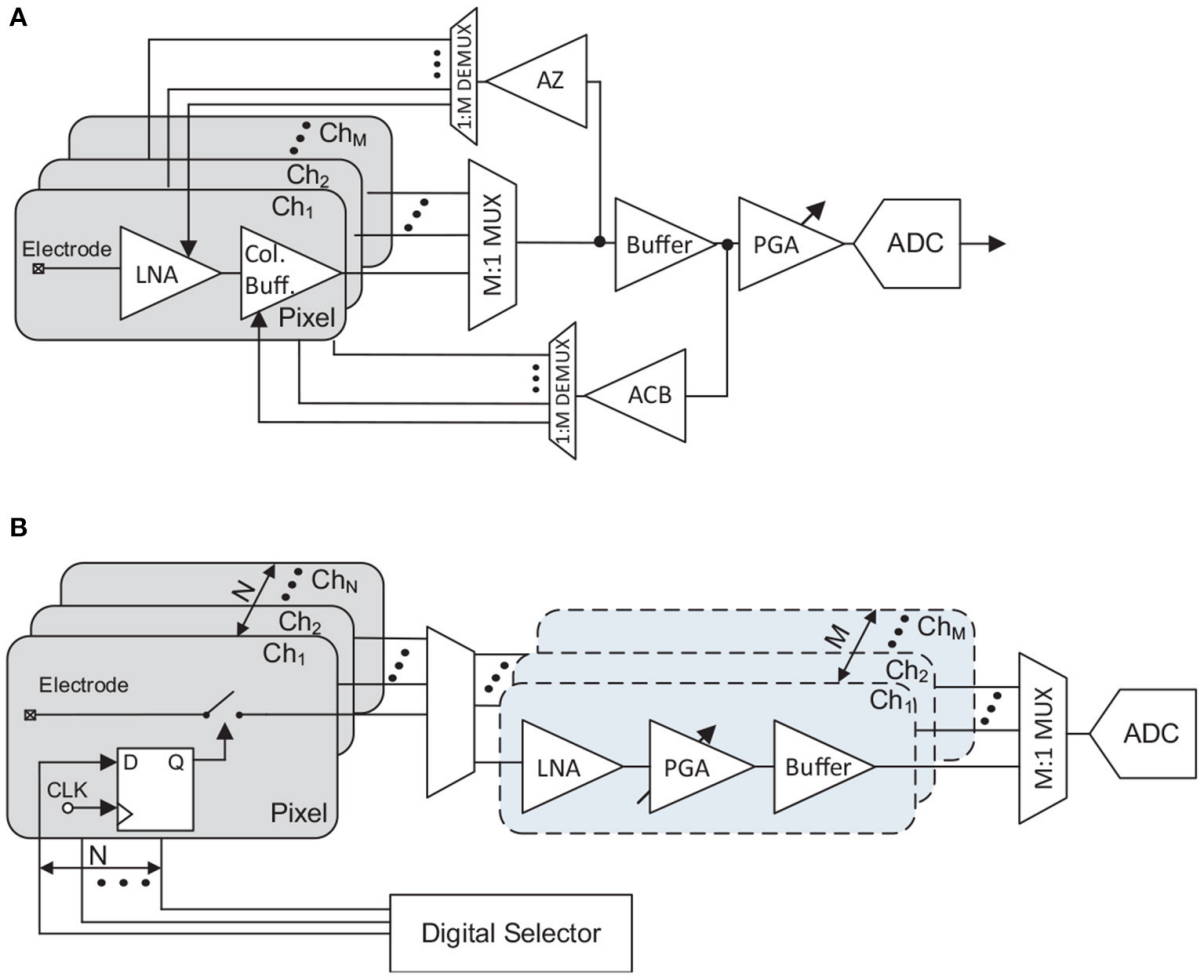
Examples of PGA sharing and switch array AFE topologies. **(A)** Block diagram of the PGA sharing AFE topology presented in Angotzi et al. (2018). **(B)** Block diagram of the switch array AFE topology presented in Dutta et al. (2019).

A similar scheme to the ADC sharing architectures involves using the same PGA and ADC for the *M* channels, as shown in Figure 8C). In the PGA sharing AFE topology, LNAs can be integrated into the same IC along with the rest of the AFE (Chae et al., 2009; Han et al., 2013; Liu et al., 2016), or along with the electrodes (Johnson et al., 2013; Angotzi et al., 2014, 2018, 2019). An interesting application example of PGA sharing with the LNA adjacent to the electrode is reported in Angotzi et al. (2018) and simplified in Figure 9A. In this architecture, the LNAs are integrated within the pixel of the neural probe and basically lie in an open-loop amplifier. To remove the DC offset without increasing the area of the pixel, an out-of-pixel autozero (AZ) amplifier is shared by all the LNAs through time-division-demultiplexing. Moreover, the column buffers are implemented in two stages: a pixel stage (with a column buffer per channel) and a base stage, i.e., not adjacent to the electrodes, which is shared by all the channels. The output of this shared buffer is fed into an amplifier (ACB). The short channel effects are then mitigated by time-division-demultiplexing the signal and feeding it into the pixel's column buffers.

### 5.3. Switch Array AFE Topology

All presented topologies allow full-frame read-out at the cost of reducing electrode density. To increase the number of electrodes, and therefore to increase the spatial resolution of the probe, a switch-matrix is integrated adjacent to the electrodes. More complex routes are used to rewire a group of electrodes to the available read-out channels (Figure 8D). This switch-matrix mainly comprises a large group of routing wires, switches, and a local memory such as a SRAM which allocates the connection status of the electrode (Frey et al., 2010), so that not so much active area is required. As in PGA sharing topologies, this architecture can also include amplifiers along with the electrodes (Huys et al., 2012; Mora Lopez et al., 2017) or just the switch-matrix (Frey et al., 2010; Ballini et al., 2014; Jun et al., 2017; Dutta et al., 2019). In this architecture, also known as static multiplexing, for *N* electrodes, the switch-matrix only selects *M* of them (with *N*>*M*) and interconnects them with the *M* read-out channels (AFEs). After the amplification stages, the signal is commonly multiplexed as in ADC sharing topologies. A simplified example of a switch array AFE corresponding to the neural probe scheme reported in Dutta et al. (2019) is shown in Figure 9B. Note that the switch-matrix incorporates memories (flip-flops) which select the electrodes to record using a digital selector integrated into the base of the neural probe. In this kind of topology, the overall form factor required defined by the number of readout channels restricts the possibilities of implementing some of the topologies of the section 3.

One of the main issues concerning these structures is the selection of the electrodes to be read and those not to be read. One widely-used solution to this involves a process which firstly records the whole electrode matrix during different time slots. The data is then processed and some groups of electrodes are prioritized to be read by applying an optimization algorithm (which could involve machine learning) based on the previously recorded signals and the main purpose of the recording. Another alternative, presented in Mora Lopez et al. (2017), divides the electrode matrix into a set of subgroups. In this proposal, the electrodes in each subgroup are selected pseudo-randomly, ensuring that all areas of the probe are recorded.

### 5.4. Time-Division-Multiplexing AFE Topology

One new trend in multi-channel neural recording topologies is to place the multiplexing at the input of a single AFE (Figure 8E) which is shared by all channels. This reduces the occupation area per channel and ignores mismatch between recording channels, potentially making the power consumption per channel lower than in conventional topologies (a further breakdown of the AFE time-division-multiplexing specifications and architectures is provided in section 6). For instance, it can be observed in Figure 6B how the TDM AFE reported in Uehlin et al. (2020) shows one of the most promising results in terms of area and noise equivalent bandwidth (NEB), which is defined as the bandwidth of a brickwall filter which produce same integrated noise power as that of the analyzed system, for this kind of topologies. The main drawback of these topologies relies on the requirement of a high-bandwidth LNA (HB LNA) to fast-multiplex all the channels, which significantly increases power consumption and in-band noise due to aliasing (Sharma et al., 2018).

## 6. Review of Time-Division-Multiplexing AFEs

One of the first reported TDM AFEs was presented in Raducanu et al. (2017). In this architecture, the TDM technique was only used for the amplifiers within the pixel, reducing the number of interconnection wires and increasing the electrode density of the neural probe. The AFE/electrode ratio, however, was still 1:1. Recently, new TDM systems have emerged with multiplexing of the whole AFE (Pérez-Prieto et al., 2019; Sharma et al., 2019; Uehlin et al., 2020). The aim of this kind of architectures is to reduce the power and area of the whole recording interface, but here two major design issues arise: noise folding and DC offset from electrodes.

### 6.1. Noise Folding in TDM AFEs

For an *M*-channel multiplexed recording device, the sampling frequency, *f*_*s*_, has to be *M* times faster than for a single channel, (*f*_*c*_ = 2 · *f*_*b*_), in order to keep the same throughput rate per channel, i.e., *f*_*s*_ = *M* · *f*_*c*_. For voltage sampling with a single pole low-pass filter response, the required IA bandwidth will therefore be given by (Sharma et al., 2018):

(6) $$\begin{array}{l} f_{BA}=\frac{\ln\left(\epsilon \right)\cdot f_{s}}{2\cdot pi}=\frac{\ln\left(\epsilon \right)\cdot f_{c}\cdot M}{2\cdot pi} \end{array}$$

where ϵ is the tolerable dynamic settling error. Thus, the NEB for the multiplexed topology will be determined by:

(7) $$\begin{array}{l} {NEB}_{TDM}=\frac{\pi }{2}\cdot f_{BA}=-f_{s}\cdot \ln\left(\epsilon \right)/4=-M\cdot f_{c}\cdot \ln\left(\epsilon \right)/4 \end{array}$$

**Figure 10:**
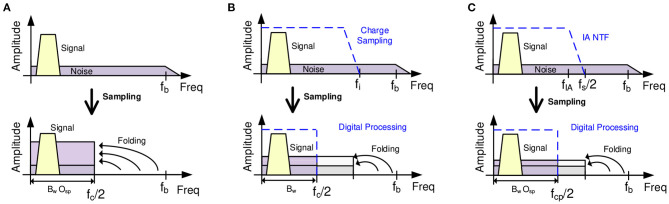
Illustration of the noise folding problem in TDM AFEs. **(A)** Noise folding problem in multiplexing circuits. **(B)** Noise folding in multiplexing circuits applying charge sampling techniques. **(C)** Noise folding in multiplexing circuits applying a narrow-band CDS architecture.

From equation 7 it can be concluded that the NEB increases proportionally with the number of channels. For APs recording, for example, the NEB in TDM AFEs is 3.5 · *M* higher than for conventional non-multiplexed AFE topologies (Sharma et al., 2018). Accordingly, the out-of-band noise components are folded-back to the baseband which largely increases the system's in-band noise due to aliasing (Raducanu et al., 2017). Figure 10A illustrates this noise folding process: at the sampling instant, all the noise components above the band of interest which are not filtered (*f*_*c*_/2 in Figure 10) are folded-back to the signal bandwidth. To solve this problem, the NEB of the AFE has to be reduced without sacrificing settling accuracy within the time allocated for channel sampling.

**Figure 11:**
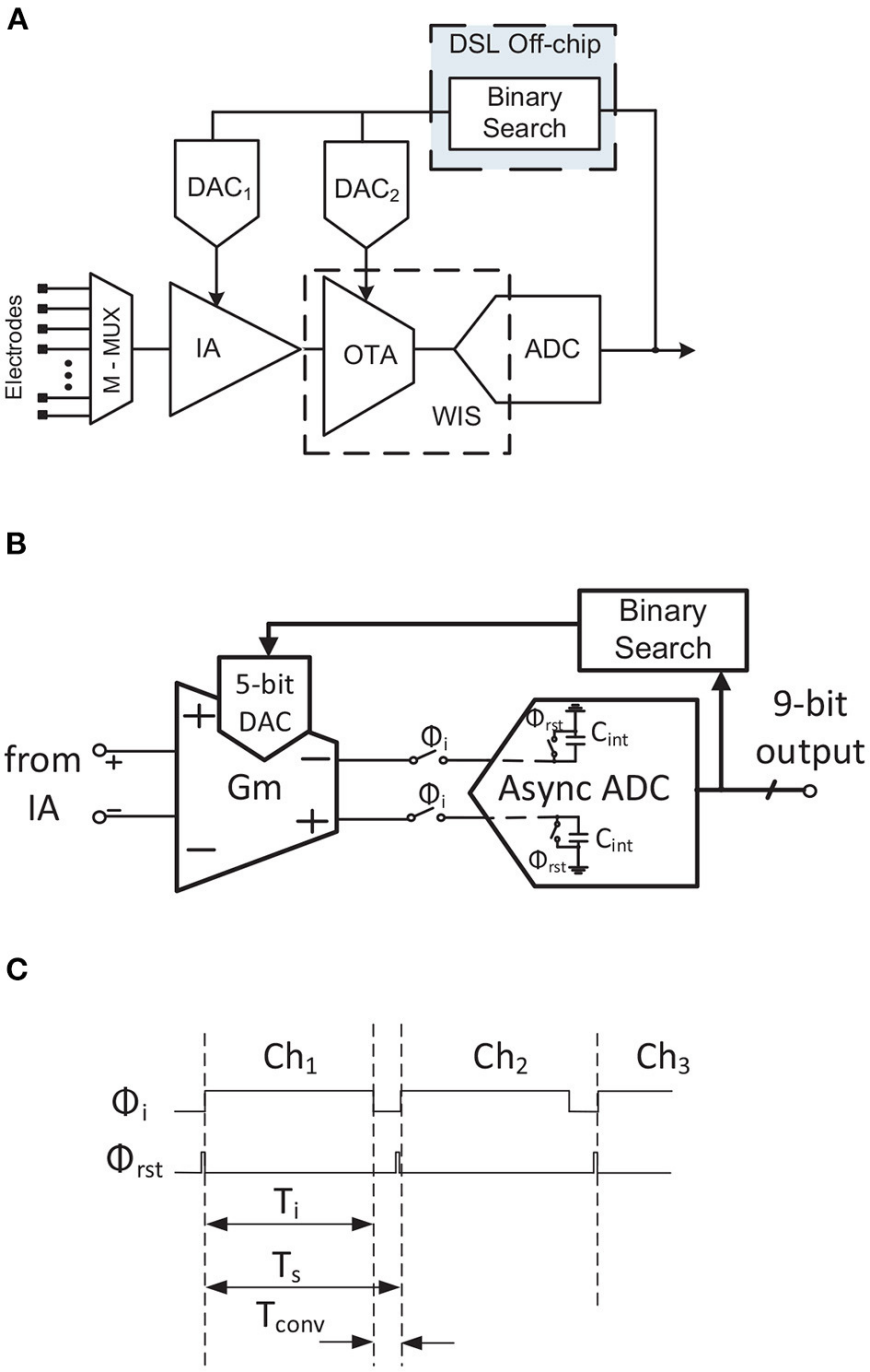
TDM AFE proposed in Sharma et al. (2019). **(A)** Block diagram. **(B)** Simplified schematic of WIS and DC Servo Loop. **(C)** Simplified timing diagram.

**Figure 12:**
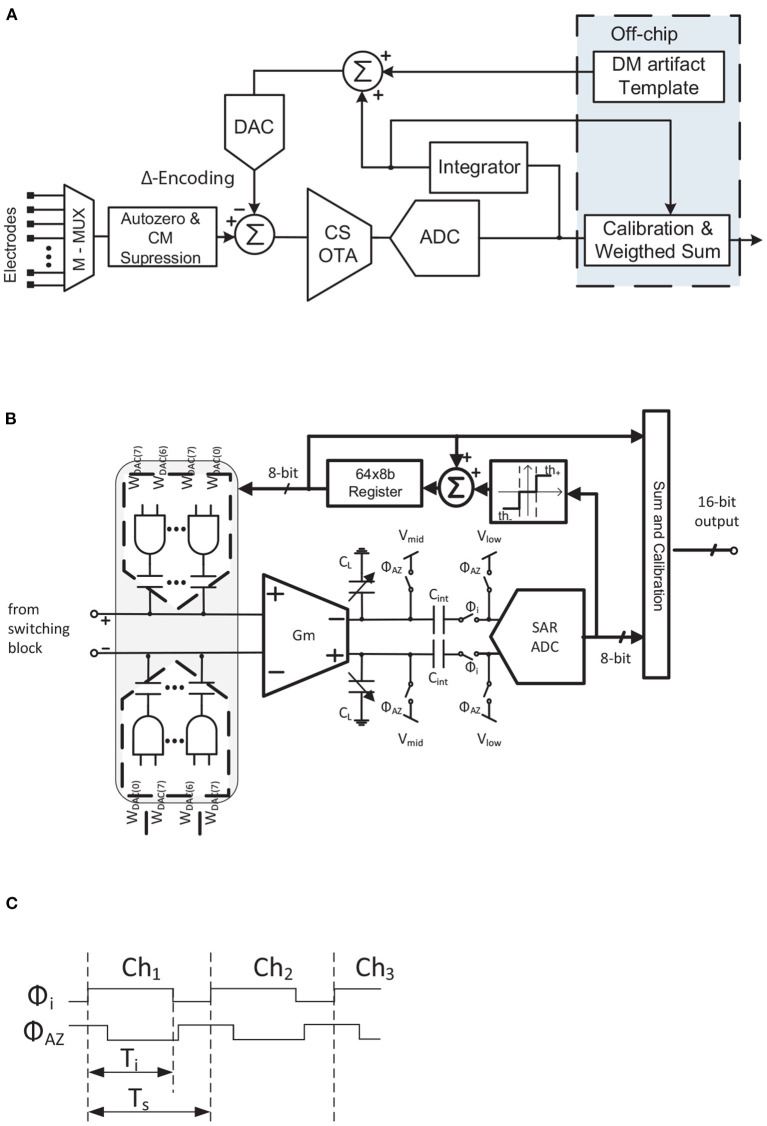
TDM AFE proposed in Smith et al. (2017) and Uehlin et al. (2020). **(A)** Block diagram. **(B)** Simplified schematic of CS amplifier and Δ-Encoding loop. **(C)** Simplified timing diagram of CS amplifier.

The first approach to coping with this problem is to apply charge-sampling (CS) instead of voltage sampling (Raducanu et al., 2017; Smith et al., 2017; Uehlin et al., 2020). The windowed integration sampling solution proposed in Sharma et al. (2018) can be considered as a kind of charge sampling. The main idea of this technique is based on integrating the signal during a period *T*_*i*_, with *f*_*i*_ = 1/*T*_*i*_ and *f*_*c*_ < *f*_*i*_ < *f*_*s*_, and then sampling the last value. High-frequency noise components are filtered (Figure 10B) according to a *sync* filter specifications, i.e., *sin*(*x*)/*x* (Gang and Jiren, 2000), reducing the noise folding effect. In terms of circuit implementation, this technique is performed by a *G*_*m*_-cell driving a sample-and-hold capacitor, *C*_*int*_, as shown in Figures 11B, 12B. The gain of this block will therefore be given by *G*_*m*_, *T*_*i*_ and *C*_*int*_ (see Table 1). However, this technique has some significant drawbacks: (i) the pole of the *sync* filter and the DC gain of the architecture are very sensitive to clock jitter (Gang and Jiren, 2000); (ii) process variations will have a high impact on the system's gain and time constant due to the employment of an open-loop structure; (iii) low-frequency noise components are not reduced; (iv) large common-mode (CM) signals could change the operating point of the *G*_*m*_ stage, which may lead to distortion or even saturation.

**Table 1:** Noise-folding aware structures comparison.

	**Architecture**	**Gain**	**Noise**	**Noise**	**Flicker**	**CMRR**	**Clock/process**	**Power**
			**filtering**	**folding**	**reduction**	**insensitive**	**variations**	**requirements**
CS / WIS	Open-Loop	$\frac{G_{m}\cdot T_{i}}{C_{int}}$	*sync*^2^	No	No	No	Weak	Low
CDS	Closed-Loop	$\frac{C_{in}}{C_{fb}}$	*sync*^2^	Yes	Yes	Yes	Robust	Medium

Another solution is to use a narrow-band correlated double sampling (CDS) scheme (Pérez-Prieto et al., 2019), Figure 13B. In this architecture, the AFE transfer function is reduced by the low-pass filter which the CDS inherently implements, as illustrated in Figure 10C. The noise in conventional CDS topologies would normally be doubled due to spectral folding, but since the sampling frequency of the CDS is higher than the CDS amplifier bandwidth, the folded noise components in the bandwidth of interest are reduced as illustrated in Figure 10C. Moreover, the flicker noise component is also palliated by the CDS scheme (Enz and Temes, 1996), while the stage power consumption retains approximately the same value as without CDS. In addition to these advantages, since the CDS is a closed-loop structure it has none of the above-mentioned gain accuracy problems, its gain being fixed by the ratio between the input capacitor (*C*_*in*_) and the feedback capacitor (*C*_*fb*_). This makes the system more robust to the influence of large CM signals.

**Figure 13:**
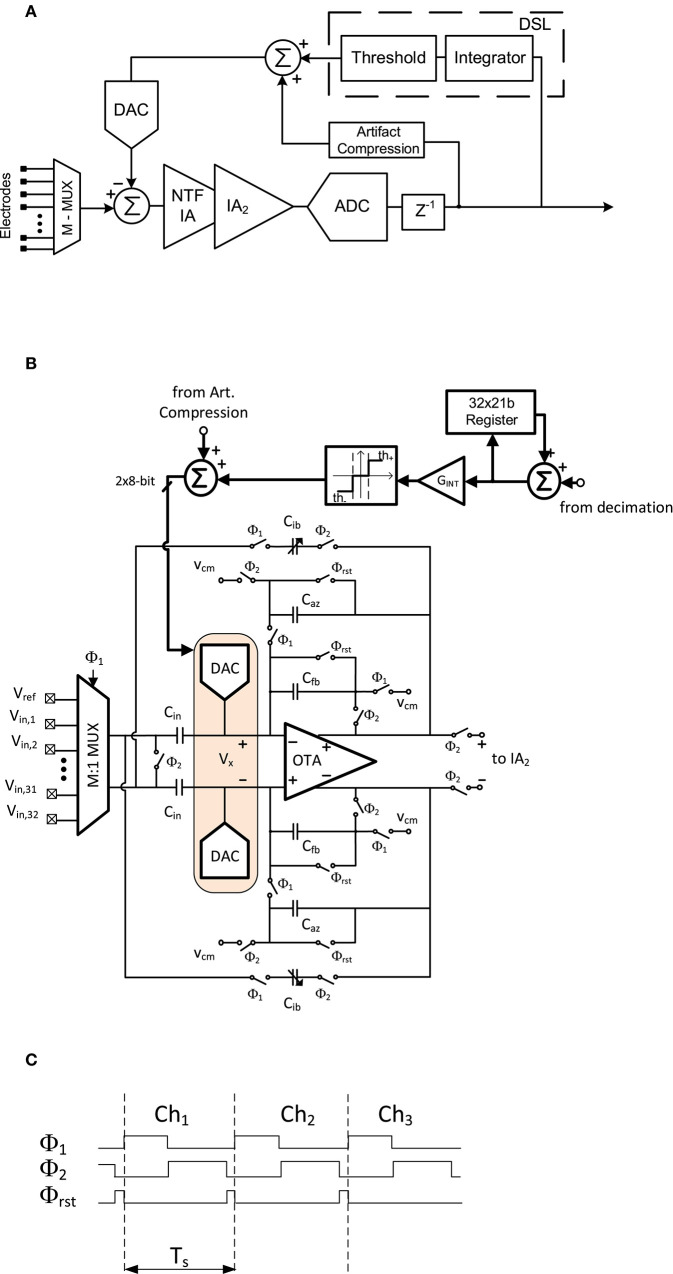
TDM AFE proposed in Pérez-Prieto et al. (2019). **(A)** Block diagram. **(B)** Simplified schematic of CDS amplifier. **(C)** Simplified timing diagram of CDS amplifier.

Table 1 briefly compares the two solutions reported for reducing noise folding in TDM AFEs. As can be seen, the main advantages of CDS over CS/WIS are related to CDS being a closed loop architecture. The highest gains, however, are achieved in CS/WIS structures without significantly increasing power consumption, whereas in CDS the power requirement for the same gain is higher.

### 6.2. DC Offset From the Electrodes in TDM AFEs

DC offset from electrodes is a recurrent problem in DC-coupled AFEs (Fan et al., 2011; Chandrakumar and Markovic, 2017; Samiei and Hashemi, 2019). In any of the presented topologies with an IA per channel, DC offset can usually be rejected using large time constant high-pass filtering. However, analog large time constant filters are not suitable in rapid multiplexing systems, since the filtering would increase crosstalk between channels and would not be fast enough to reject the DC offset from each channel. One solution to this problem could be to limit the gain of the AFE and to increase the resolution of the ADC. However, this extra resolution, together with the high sample rate required for multiplexing, would make the ADC unsuitable for low-power designs.

High-pass filtering the signal through a mixed-signal loop has been adopted as an alternative approach to palliating the DC offset problem in DC-coupled topologies (Muller et al., 2015; Bagheri et al., 2017). In this method, a sub-Hz finite-impulse-response (FIR) or infinite-impulse-response (IIR) filter is fed into the input of the AFE by a digital-to-analog converter (DAC). While the filter can be designed to not penalize the system's power consumption and occupation area, the required DAC resolution has to be high enough not to increase the noise at the input of the AFE. The number of bits required will be determined by the resolution of the ADC, the overall gain through the signal path, and the IRN of the AFE. In most practical cases, a DAC of more than 16-bits is required, which strongly compromises the form factor specification of the neural recording AFE. One proposed solution for implementing this high-resolution DAC is to employ a ΔΣ modulator (Muller et al., 2015; Bagheri et al., 2017). However, this method is not feasible for TDM AFEs because the required oversampling frequency will be multiplied by *M*, and this will significantly impact the power consumption of the digital part of the IC. An alternative to a high-resolution DAC would be to use a binary search algorithm (Sharma et al., 2019) which initially computes the DC offset codes for each channel and retains the correction values until a threshold condition occurs. At that instant, the binary search recalculates the correction value for each channel. By applying this method, DC offset drifts are palliated without increasing the IRN of the AFE. On the other hand, although the system range is ensured, there will be residual offset at the output of the AFE. This will have to be filtered in the digital domain.

Another proposed solution is based on working with Δ-signals (as illustrated in Figures 4C–E). In this approach, the system tracks differences between successive samples, high-pass filtering the input signal. The signal then has to be reconstructed in the digital domain using an integrator/accumulator. This technique can be transferred to TDM AFE topologies by employing registers to store the previously sampled value of each channel. One example of a TDM AFE which exploits this technique is reported in Smith et al. (2017); Uehlin et al. (2020).

### 6.3. Comparison of TDM AFE Architectures

Despite their promising results, TDM AFE topologies have not to date been researched in depth. In this subsection, three reported TDM AFE architectures are detailed. Block diagrams of these neural AFEs are shown in Figures 11A, 12A, 13A.

The first architecture, reported in Sharma et al. (2018) and Sharma et al. (2019), is shown in Figure 11A. The IA comprises a capacitive feedback single-stage cascaded OTA. An open-loop OTA is employed as a *G*_*m*_-cell along with the SAR ADC capacitors to implement the WIS filter and to further amplify the signal (Figure 11B). This reduces the high-frequency noise components from the IA. The timing diagram of this operation is shown in Figure 11C. It can be seen that the integration period, *T*_*i*_, lasts for most of the sampling period *T*_*s*_. After that, before the capacitors of the ADC (ϕ_*rst*_) are reset and the input channel is changed, the conversion phase, *T*_*conv*_, takes place for only 11% of the sampling period. This short-time conversion is carried out by an asynchronous converter. To remove the DC offset, a binary search algorithm is implemented externally by a Python script. This algorithm recomputes the 9-bit code each second to palliate the input DC offset. This is fast enough to compensate DC drifts at the input. The code is divided into 4-bits for DAC_1_ and 5-bits for DAC_2_ and maximizes the useful dynamic range of the system while reducing the ADC requirements. It should also be mentioned that both DACs are embedded in the amplifier structure.

A Δ-Encoded TDM AFE was first presented in Smith et al. (2017) and further developed in Uehlin et al. (2020) (Figure 12A). In this architecture, after the input multiplexer, an input switching scheme consisting of a set of switches with a couple of input capacitors performs two main functions: (i) autozeroing the inputs to reduce crosstalk between adjacent channels; and (ii) largely suppressing the CM signals. An 8-bit capacitive DAC connected to the input node of the OTA then carries out the Δ-operation by subtracting the signal value previous to the present value. This also minimizes the DC offset, improving the system's dynamic range. Afterwards, the Δ-signal is amplified by a charge-sampling amplifier consisting of an open-loop *G*_*m*_-cell and capacitors *C*_*L*_ (Figure 12B). Note that the value of *C*_*L*_ is variable, mainly to set the gain of the charge-sampling topology and to palliate the ϕ_*i*_ clock variations. A timing diagram of this charge-sampling block in normal recording mode is shown in Figure 12C. Firstly, the signal is integrated during ϕ_*i*_. Then, in the ϕ_*AZ*_ phase the capacitors are reset. Once the signal is converted by an 8-bit SAR ADC, it can follow two paths: (i) through the mixed-signal loop to perform the encoding technique and (ii) to the output. The first step of the mixed-signal loop is a user-programmable threshold block which determines the update quantity of the tracking signal. The update values, which can be −1, 0, or +1, are added to the previous tracking value. A 64x8-bit register stores the previous values of the correction signal for each channel. This register, together with the tracking update, performs an integration loop. The output signal from this loop feeds the DAC and is also scaled and added to the ADC output in order to reconstruct the signal. The output code thereby increases its resolution from 8 to 16 bits.

Another approach to TDM AFE is shown in Figure 13A (Pérez-Prieto et al., 2019). In this architecture, both IAs are implemented using narrow-band CDS architectures to filter the flicker and high-frequency noise components of the circuit and to provide a robust closed-loop structure against CM interferences. The scheme of the first IA is shown in Figure 13B. An 8-bit capacitive DAC is connected to each input virtual ground node of the IA, *V*_*x*_. These DACs close the mixed-signal loop which, in addition to rejecting the DC offset, also implements an artifact compression technique, thus increasing the dynamic range of the circuit. The input impedance of the AFE is boosted by a couple of capacitors, *C*_*ib*_, included in the CDS loop (Fan et al., 2011). The timing diagram of this stage is shown in Figure 13C. Before reading the input of the multiplexer, the feedback capacitors are reset in order to reduce crosstalk between adjacent channels. The signal is then amplified and flicker-reduced in phase ϕ_2_. It is then oversampled and, after analog-to-digital conversion, filtered and decimated. The resolution of the signal is thus increased from 10 to 14 bits. In the mixed-signal loop, the DSL mainly comprises an integrator, the gain of which sets the sub-Hz cutoff frequency. This integrator is voltage-triggered so as not to produce input oscillations.

### 6.4. Comparison of State-of-the-Art TDM and High-Performance AFEs

Table 2 shows a final comparison between TDM AFEs and the high-performance neural AFEs presented during section 3. Due to the fact that each presented AFE topology comprises several and different works, this comparison has been carried out by employing the lowest and highest reported values for each topology. Thus, it is worth observing how TDM AFEs provide the lowest values in terms of occupation area without significantly penalizing the rest of the AFEs' specifications. This Table corroborates the comparisons of the state-of-the-art previously presented in section 3.

**Table 2:** Comparison of high-performance AFEs (range of values).

**AFE topology**	**Continuous-time**	**Chopper-stabilized**	**Chopper-based ΔΣ**	**ΔΣ**	**Time/frequency**	**TDM**
					**based**	**Uehlin et al., 2020**
Power/channel	[0.0015^*d*^–114.8^*c*^]	[0.017^*j*^–2160^*i*^]	[0.8^*p*^–9.9^*n*^]	[0.63^*s*^–4.79^*r*^]	[3.2^*y*^–21^*x*^]	2.98
(μ*W*/ch)						
IRN normalized	[0.0068^*b*^–1.354^*d*^]	[0.0038^*i*^–0.7518^*l*^]	[0.0443^*p*^–0.2656^*q*^]	[0.0492^*r*^–0.0716^*t*^]	[0.0325^*x*^–0.639^*w*^]	0.0884
($\mu V_{rms}/\sqrt{Hz}$)						
Area/channel	[0.0098^*f*^–0.26^*e*^]	[0.017^*k*^–0.81^*j*^]	[0.018^*n*^–0.55^*q*^]	[0.013^*u*^–0.023^*t*^]	[0.01^*y*^–0.135^*v*^]	0.0023
(mm^2^/ch)						
Input impedance	[4^*b*^–61^*a*^]	[1^*i*^–5400^*h*^]	[20^*o*^–1000^*n*^]	[1^*s*^–1000^*r*^]	[50^*w*^–∞^*v*^)	92
(MΩ)						
NEF	[1.07^*g*^–19.4^*c*^]	[0.86^*m*^–126.7^*l*^]	[1.81^*p*^–26.03^*q*^]	[2.86^*u*^–5.99^*r*^]	[3.33^*z*^ –57.61^*w*^]	2.35

a *Leene and Constandinou, 2017*;

b *Zhang et al., 2013*;

c *Mohseni and Najafi, 2004*;

d *Harpe et al., 2015*;

e *Rai et al., 2009*;

f *Kuhl and Manoli, 2015*;

g *Shen et al., 2018*;

h *Ha and Yoo, 2016*;

i *Jiang et al., 2019*;

j *Chen et al., 2015*;

k *Chandrakumar and Markovic, 2017*;

l *Xu et al., 2014*;

m *Mondal and Hall, 2020*;

n *Bagheri et al., 2017*;

o *Chandrakumar and Markovic, 2018*;

p *Kim et al., 2018*;

q *Bang et al., 2018*;

r *Muller et al., 2012*;

s *Kassiri et al., 2017*;

t *O'Leary et al., 2018*;

u *Muller et al., 2015*;

v *Jiang et al., 2017*;

w *Mohan et al., 2017*;

x *Tu et al., 2017*;

y *Huang et al., 2018*;

z *Zhao et al., 2020*.

## 7. Discussion and Future Works

This work has presented a review of recording techniques for high-channel-count, densely-spaced microelectrode arrays. Two of the main concerns when increasing the number of read-out channels in neural recording devices are the occupation area and the power consumption of the silicon-based signal conditioning circuitry. Although the design effort has to be focused on these two factors, other significant neural AFE specifications such as low noise contribution, low crosstalk between channels and high input impedance also have to be satisfied.

Firstly, the issue of whether or not to employ active electrode-AFE interfaces was introduced. With regard to crosstalk, the reported analysis in Du et al. (2009); Seidl et al. (2012); Lopez et al. (2014) has shown how placing the amplifier adjacent to the electrodes significantly minimizes crosstalk between the interconnection wires. However, the power consumption and occupation area of the amplifier are severely limited by the heating and the form factor of the active area. The noise contribution of the active electrode-AFE interface will therefore be larger than for an amplifier placed on the base of the neural probe. With regard to thermal noise, the power constraints of these interfaces depend on the device employed, and this improves the design flexibility to reduce the thermal noise floor. On the other hand, flicker noise is larger for the active interfaces due to the small size of the employed amplifier. The main consideration when using or not using active electrode-AFE interfaces is therefore the reported crosstalk-flicker noise tradeoff.

High-performance neural AFE topologies have been disclosed and briefly introduced and compared. Herein, one commonly employed method for reducing the number of recording blocks is the TDM technique. This paper proposes a classification of neural recording architectures into five different topologies based on the location of the multiplexer in the signal path. Over the last few years, non-multiplexed AFE topologies have been consolidated as one of the best techniques in terms of power consumption and occupation area thanks, among other things, to their design flexibility. Moreover, different schemes of non-multiplexed architectures have been introduced, demonstrating different alternatives for implementing these topologies without penalizing neural AFE specifications. Also, novel TDM AFEs have demonstrated the capability of multiplexing at the AFE input to reduce area and power more than in conventional ADC or/and PGA sharing topologies. Although some strategies, such as charge sampling, have been reported to filter high-frequency noise components, the trade-off between the number of multiplexed channels and the noise increment due to aliasing has to be taken into account in the design process. Furthermore, no TDM structures have been reported with more than 64 channels. This work also provides a state-of-art comparison illustrating how non-multiplexed AFEs and TDM AFEs are generally reported to offer the best performance, while switch array AFEs have the largest number of input channels.

Future advances in neural recording techniques should follow the trends mentioned above. Although not mentioned during this work, one of the main problems associated with these techniques is the processing and transmission of data. Increasing the number of channels considerably increases the amount of data to be processed and transmitted. This increases consumption in the digital part of neural recording systems, making it comparable with that of the analog part. Moreover, as these systems are intended for long-duration implants, the amount of data to be stored could be too big. New techniques for data compression and feature extraction must therefore emerge to address these problems.

## Author Contributions

NP-P and MD-R conceptualized the study, processed the data and wrote and reviewed the article. All authors contributed to the article and approved the submitted version.

## Conflict of Interest

The authors declare that the research was conducted in the absence of any commercial or financial relationships that could be construed as a potential conflict of interest.
